# Acupuncture Combined With Emotional Therapy of Chinese Medicine Treatment for Improving Depressive Symptoms in Elderly Patients With Alcohol Dependence During the COVID-19 Epidemic

**DOI:** 10.3389/fpsyg.2021.635099

**Published:** 2021-05-26

**Authors:** Fazheng Zhao, Xin Tong, Changqing Wang

**Affiliations:** ^1^Nanjing Medical University, Nanjing, China; ^2^Heilongjiang University of Chinese Medicine, Harbin, China

**Keywords:** acupuncture, emotional therapy, elderly alcohol dependence, depressive symptoms, COVID-19

## Abstract

**Objective:** We aimed to analyze the characteristics and psychological mechanism of depressive symptoms in elderly patients with alcohol dependence under the COVID-19 epidemic and to observe the effect of acupuncture combined with emotional therapy of Chinese medicine treatment on depressive symptoms in elderly patients with alcohol dependence.

**Methods:** Sixty patients were randomly divided into two groups. One group was treated by a set of emotional therapy of Chinese medicine treatment for 12 weeks (control group). One group was treated by a set of acupuncture combined with emotional therapy of Chinese medicine treatment for 12 weeks (treatment group). We compared the curative effect between the control group and the treatment group, the mean alcohol consumption, the SF-36 scores before and after treatment, and the scores of Hamilton Depression Scale before and after treatment of 3, 6, and 9 weeks.

**Results:** Based on the cognitive behavior model, the characteristics and psychological mechanism of depression in elderly patients with alcohol dependence under the COVID-19 epidemic situation were summarized. The total effective rate of the control group was 60%, and that of the treatment group was 100% (*p* < 0.05). The alcohol consumption of the patients in each group decreased significantly after treatment (*p* < 0.05), and there was no significant difference in alcohol consumption between the treatment group and the control group (*p* > 0.05). After 12 weeks of treatment, there were significant differences in PF, RF, physical pain, general health status, energy, and mental health between the treatment group and the control group (*p* < 0.05). Before and after treatment, there were significant differences in PF, RF, physical pain, general health, energy, emotional function, and mental health (*p* < 0.05) of the treatment group. The PF, energy, and mental health of the control group were significantly different before and after treatment (*p* < 0.05). There was no significant difference between the treatment group and the control group in the scores of Hamilton Depression Scale before treatment. There was significant difference between the treatment group and the control group in the scores of Hamilton Depression Scale at 3, 6, and 9 weeks after treatment.

**Conclusion:** Attention, cognition, emotion, behavior, and physical response reinforce each other, creating a vicious cycle that reinforces and sustains the depressive symptoms of elderly alcohol dependence under the COVID-19 epidemic, and acupuncture combined with emotional therapy of Chinese medicine treatment for improving the depressive symptoms of elderly alcohol dependence during the epidemic period of COVID-19 has a brilliant therapeutic effect.

## Introduction

Recently, the United Nations issued a policy brief on “the epidemic situation of COVID-19 and mental health,” which pointed out that the epidemic situation not only damages people's lives and physical health but also has a serious impact on people's psychology and spirit, and causes the associated physical and mental illness (National Board of Health, 2020). The continuation of the epidemic has had an enormous impact on the physical and mental health of the elderly in particular. Old people usually live alone, they have a lack of communication with others, and the rate of serious disease and death rate is higher, which may cause a series of negative emotions and even psychological problems (Zhang et al., 2020). During the epidemic period of COVID-19, old people tend to interpret both ordinary and unusual physical sensations in a negative way, thereby giving rise to excessive concern and concern for their physical well-being, resulting in individual suffering and substance abuse; persistent health concerns may increase the risk of alcohol dependence (Salkovskis and Warwick, 1986; Fink et al., 2010; Sunderland et al., 2013). Alcohol dependence is a series of special physiological and psychological reactions caused by excessive and repeated drinking. During the epidemic period of COVID-19, the elderly patients with alcohol dependence showed cravings for alcohol due to their bad physical and mental condition and the forced experience of drinking alcohol frequently, which ran through the whole dependence process; it is characterized by withdrawal syndrome, relapse, and tolerance. The patients show severe depressive symptoms in the process of alcoholism and abstention. The important psychological cause of the disease is anxiety about their health under emergency conditions (Zheng et al., 2005; Tyrer et al., 2020). The depression symptom perplexity causes the patient's psychological burden to aggravate, which, in turn, causes the failure to stop drinking. In the treatment of alcohol dependence, drug therapy is often used. Long-term use leads to the injury of liver, kidney, and other organs. The patients suffer great economic and economic burden and, at the same time, bring serious injury to the body. Acupuncture therapy under the guidance of the basic theory of traditional Chinese medicine takes human physiology and psychology as an organic whole and has the advantage of treating body and mind together. In the course of clinical treatment, acupuncture therapy not only fully considers the influence of biological factors on the disease but also pays more attention to the role of various psychological factors on the outcome of the disease. Acupuncture therapy is a safe and effective method for clinical treatment of physical and mental diseases by distinguishing the physical and mental characteristics of patients and treating patients individually. Emotional therapy of Chinese medicine treatment and modern cognitive behavior therapy (CBT) have the same idea; the intervention on health care is very effective and the curative effect is lasting (Olatunji et al., 2014; Tyrer et al., 2014; Cooper et al., 2017; Axelsson and Hedman-Lagerlöf, 2019), and it is helpful to alleviate health care and improve the overall health condition and reduce the economic costs associated with health concerns (Morriss et al., 2019). This study is based on the holistic view of mind and body of traditional Chinese medicine (TCM) and, on the basis of dialectical analysis of psychosomatic characteristics of elderly alcohol dependence and depressive symptoms under the COVID-19 epidemic, carries out acupuncture treatment and integrates TCM emotional therapy into it to improve the treatment of elderly patients with alcohol dependence.

## Characteristics and Mechanism

The characteristics of depressive symptoms in elderly patients with alcohol dependence under the COVID-19 epidemic are as follows: (1) Cognitive characteristics: (i) Disease Belief: that they have developed COVID-19; (ii) Disease Preemption Concept: the idea and picture of COVID-19 appeared repeatedly; (iii) A heightened awareness of bodily sensations and changes. (2) Somatic characteristics: (i) Anxiety-related somatic reactions: increased heart rate; (ii) A slight bodily change or sensation that is distorted (as a slight fluctuation in body temperature or a dry tickle in the throat). (3) Emotional characteristics: (i) Fear of having developed COVID-19; (ii) Fear of future infection with COVID-19; (iii) Fear or anxiety about exposure to stimuli associated with neocoronary pneumonia. (4) Behavioral characteristics: (i) Checking and confirming: such as checking the body again and again, asking for a nucleic acid test, spending a lot of time searching, and looking up information about COVID-19; (ii) Fear of catching COVID-19 in the future: stocking up on protective materials and food, washing repeatedly, sterilizing, overprotecting, and avoiding the stimuli associated with COVID-19 (such as staying indoors, working, or taking time off from school).

The cognitive behavior model of health anxiety was first proposed by Salkovskis et al. (Salkovskis and Warwick, 1986), on the basis of which the following researchers proposed a CBT-based integrated model of health anxiety. The alcohol-dependent elderly patients with depressive symptoms during the epidemic period of COVID-19 have potentially poor health perceptions (beliefs) that can be activated by different events, such as disease-related news reports or slight changes in somatic sensation. When triggered, these poor health perceptions can lead to an individual's heightened awareness of any bodily sensations or changes that could indicate illness, and a disastrous interpretation of perceived bodily sensations or changes, triggering health anxiety; these health anxiety triggers can lead to behavioral and physical changes, further reinforcing poor health perceptions and increasing attention to changes in body perception, leading to more pronounced health anxiety and creating a vicious cycle.

In the case of COVID-19, the elderly may be filled with tension in their daily lives when they see information about the epidemic in the media, whether there are confirmed cases in their city or near their place of residence. In this context, the individual's own underlying distorted beliefs about health are activated, becoming particularly alert to information and cues related to COVID-19, and paying close attention to their own physical responses and a disastrous explanation of the slight physical sensations and changes and distorted perceptions such as “I have a little tickle in my throat that I have been infected by COVID-19 virus” and “It is a worldwide pandemic and I must be infected,” causing health concerns that lead to a range of non-adaptive behaviors and physical responses (Warwick and Salkovskis, 1990). While non-adaptive behaviors, such as repeated hand washing and hand sanitizing, alleviate anxiety in the short term, in the long term, they confirm and reinforce an individual's perception of poor health (Marcus et al., 2007). In anxious situations, the presence of somatic responses such as a scratchy throat or small fluctuations in body temperature increases the individual's focus on somatic responses and can lead to an increase in health anxiety, thereby reinforcing alcohol-dependent behavior. Thus, attention, cognition, emotion, behavior, and physical response reinforce each other, creating a vicious cycle that reinforces and sustains the depressive symptoms of alcohol dependence in elderly patients under the COVID-19 epidemic.

## Information and Method

In the following study, 60 elderly alcohol-dependent patients with depressive symptoms hospitalized in a class A tertiary hospital in Heilongjiang province from May 2020 to October 2020 were selected for therapy. Patients were randomly divided into two groups. One group was treated by a set of emotional therapy of Chinese medicine treatment for 12 weeks (control group). One group was treated by a set of acupuncture combined with emotional therapy of Chinese medicine treatment for 12 weeks (treatment group). There were 60 elderly patients with alcohol dependence and depressive symptoms, including 38 males and 22 females, aged 60–75 years. History of drinking ranged from 12 to 38 years (average, 23.5 years). The mean body weight was 63 ± 6.1 kg, and average daily pure alcohol consumption was 38.69 ± 15.31 g, drinking four to seven times per day. Patients who had signed an informed consent form were randomly divided into two groups, regardless of age or alcohol consumption, and the groups were comparable. All the selected patients underwent physical examination without family and personal history of physical and mental diseases. The depression symptoms occurred after drinking. Patients met the World Health Organization's DIAGNOSTIC CRITERIA FOR ALCOHOL DEPENDENCE: (1) uncontrollable urge to drink; (2) a daily regular drinking pattern; (3) the need to drink more than any other activity; (4) an increase in alcohol tolerance; (5) recurrent withdrawal symptoms; (6) only continued drinking may eliminate withdrawal symptoms; and (7) withdrawal often leads to relapse (Zhang, 1993). All patients met CCMD-3 criteria for the diagnosis of alcohol-induced depression, with the following two (or more) symptoms: insomnia, heart palpitations, gastrointestinal symptoms, chronic pain, memory loss, depression, anxiety, and other symptoms (Zhang, 1993). Symptoms appear for as short as 3 months and as long as 6 years. The scores of 60 patients were 16 and 27, respectively, with an average score of 22.34. The patients were treated with TCM emotional therapy (control group) and acupuncture combined with TCM emotional therapy (treatment group) for 12 weeks.

### The Method of Treatment

The treatment group is subjected to acupuncture combined with Chinese medical emotional therapy. The control group is subjected to Chinese medical emotional therapy. The methods of acupuncture are taking Baihui and Neiguan points (alternating left and right, unilateral selection), flat reinforcing and reducing manipulation, and Zusanli moxibustion. The needle is kept for 30 min and is done once every 15 min, two times per week, and eight times for the course of treatment, with a total of three courses of treatment. There are four steps of Chinese medical emotional therapy based on the cognitive behavior therapy.

Chinese medical emotional therapy is based on the idea of helping elderly patients with alcohol dependence to express their emotions and guiding them with the idea of benefiting their mental and physical health. According to the “The Medical Classic of the Yellow Emperor,” it is human's instinct to seek benefit and avoid harm. The key to treatment is to understand the causes of the disease, to be aware of the detrimental effects of unhealthy behaviors on health, and to develop individualized treatment plans for elderly patients with alcohol dependence, poor compliance behavior, non-cooperation, and a high recurrence rate. All-day drinking with no self-control is due to emotional disorders and depression for a long time (to drink away sorrow and abnormal pain). The first step of Chinese medical emotional therapy is to point out the harm of the disease according to the individual condition of the patient and stimulate the patient's psychology of seeking treatment. The second step is to make the patient feel understood on the basis of the first step, a sense of belonging, and help the patient to vent through talking. The third step is to guide the elderly patient's cognition in the direction beneficial to the treatment of the disease. The fourth step is in-depth treatment, to further help the patient to remove emotional, behavioral, and physical disorders and obtain good results. The treatment plan is treatment once a month and continuous treatment for 3 months.

### Criteria for Evaluation of Efficacy

The SF-36 scale, which is widely used to assess alcohol dependence, was used to assess the score (Daeppen et al., 1998; Zhang, 2005; Luquiens et al., 2012). The final SF-36 score formula was: Final SF-36 Score = 100% (actual initial score – theoretical minimum initial score)/(theoretical maximum initial score – theoretical minimum initial score). Before treatment and 3, 6, and 9 weeks after treatment, patients were scored for depression using the Hamilton Depression Scale, and then the efficacy was evaluated according to the description of symptoms in the Chinese Medicine Syndrome Questionnaire for alcohol dependence, clinical recovery (Tong, 2012; Wang, 2014): symptoms and signs disappeared or basically disappeared; Effective: symptoms and signs are improved; Invalid: symptoms and signs are not significantly improved, or even worse.

### The Method of Statistics

The measurement data were expressed by mean ± standard deviation and were statistically processed by SPSS22.0 software.

## Results

### Evaluation of the Curative Effect Between the Control Group and the Treatment Group

The total effective rate of the control group was 60%, and that of the treatment group was 100% (*p* < 0.05). The results showed that the curative effect of acupuncture combined with TCM emotional therapy was obviously better than that of emotional therapy of the Chinese medicine treatment group. Acupuncture can adjust the circulation and metabolic function of human body. This body-mind approach works better than that of emotional therapy of the Chinese medicine treatment alone. Clinically, patients with elderly alcohol dependence may experience impaired glucose metabolism and energy supply. Acupuncture may improve the physiological and psychological state of elderly patients with alcohol-related syndrome from the perspective of regulating human glucose metabolism. However, the mechanisms of the treatment are still needed to be studied in the future (Table 1).

**Table 1:** Comparison of curative effect between the control group and the treatment group examples (%).

**Groups**	***n***	**Full recovery**	**Effective**	**Null and void**	**Total efficiency**
CG	30	9	9	12	60.0
TG	30	16	14	0	100.0^*^

* *p < 0.05*,

*the treatment group was compared with the control group*.

### Comparison of Daily Average Alcohol Consumption Between the Control Group and the Treatment Group Before and After Treatment

The average daily alcohol consumption before and after treatment was converted into grams of pure alcohol. The results showed that the alcohol consumption of the patients in each group decreased significantly after treatment (*p* < 0.05), and there was no significant difference in alcohol consumption between the treatment group and the control group (*p* > 0.05). The results showed that both groups could reduce the average daily alcohol consumption, indicating that the treatment achieved the desired effect, but how to make TCM emotional therapy play a greater role in the course of treatment should be further discussed in future research (Table 2).

**Table 2:** Comparison of daily average alcohol consumption before and after treatment.

**Groups**	**Average daily alcohol consumption (g) 1 week prior to treatment**	**Average daily alcohol consumption (g) 1 week after treatment**
CG	32.16 ± 8.80	26.21 ± 7.46^*^
TG	33.12 ± 7.21	27.25 ± 8.11^*^^▴^

* *p < 0.05, comparison between pre-treatment and post-treatment*.

▴ *p > 0.05, the treatment group was compared with the control group*.

### Comparison of SF-36 Scores Between the Control Group and the Treatment Group Before and After Treatment

After 12 weeks of treatment, there were significant differences in PF, RF, physical pain, general health status, energy, and mental health between the treatment group and the control group (*p* < 0.05). Before and after treatment, there were significant differences in PF, RF, physical pain, general health, energy, emotional function, and mental health (*p* < 0.05) of the treatment group. The PF, energy, and mental health of the control group were significantly different before and after treatment (*p* < 0.05). The results showed that acupuncture combined with TCM emotional therapy can increase the therapeutic effect (Table 3).

**Table 3:** Comparison of SF-36 scores between two groups before and after treatment.

**Groups**		**PF**	**RF**	**Somatic pain**	**General health condition**
TG	Pre-treatment	65.0 ± 4.0	51.3 ± 3.2	80.5 ± 4.1	64.2 ± 4.1
	Post-treatment	73.0 ± 3.2^*^^▴^	67.2 ± 4.2^*^^▴^	90.3 ± 3.2^*^^▴^	76.7 ± 5.4^*^^▴^
CG	Pre-treatment	64.1 ± 3.3	50.7 ± 2.5	81.1 ± 3.8	63.1 ± 4.0
	Post-treatment	66.8 ± 4.6^*^	52.8 ± 3.0	81.8 ± 4.6	65.4 ± 3.2
**Groups**		**Energy**	**Social function**	**Emotional function**	**Mental health**
TG	Pre-treatment	61.3 ± 5.3	67.0 ± 6.2	75.2 ± 5.3	57.1 ± 3.5
	Post-treatment	74.1 ± 4.0^*^^▴^	69.3 ± 4.1	79.7 ± 5.2^*^	68.0 ± 4.5^*^^▴^
CG	Pre-treatment	60.6 ± 3.9	66.1 ± 4.7	75.6 ± 5.0	57.6 ± 5.8
	Post-treatment	63.8 ± 4.6^*^	67.8 ± 2.4	78.0 ± 3.1	60.8 ± 3.1^*^^▴^

* *p < 0.05, comparison between pre-treatment and post-treatment*.

▴ *p < 0.05, the treatment group was compared with the control group*.

### Comparison of Hamilton Depression Scale Scores Between Control Group and Treatment Group Before and After Treatment

There was no significant difference between the treatment group and the control group in the scores of Hamilton Depression Scale before treatment. There was significant difference between the treatment group and the control group in the scores of Hamilton Depression Scale at 3, 6, and 9 weeks after treatment. Under the huge epidemic disaster and stress, the elderly alcohol-dependent patients need more personalized psychological counseling in order to effectively alleviate the depression (Table 4).

**Table 4:** Comparison of Hamilton Depression Scale scores.

**Groups**	***n***	**Pre-treatment**	**3 weeks later**	**6 weeks later**	**9 weeks later**
CG	30	31.43 ± 3.24	27.33 ± 3.40	19.21 ± 2.24	16.43 ± 3.51
TG	30	29.30 ± 3.11	23.54 ± 3.17^*^	14.43 ± 2.30^*^	11.48 ± 3.01^*^

* *p < 0.01, the treatment group was compared with the control group*.

## Discussion

Through clinical observation, it was found that the depression of the elderly patients with alcohol dependence during the epidemic period of COVID-19 was caused by not only emotional stimulation but also the loss of confidence and determination after the failure of abstention, a violent mood change. Because of the long-term effects of alcohol, metabolic disorders and abnormal secretion of neurotransmitters in the body cause a series of chronic pain, dizziness, and other physical discomfort and depression. Elderly patients with alcohol dependence and depression are often depressed due to emotional injury. Acupuncture can adjust the patients' emotional disorder by regulating the circulation of the body. Human's psychological activity and physiological activity are a pair of main contradictory movements in normal life. They interact and condition each other. Psychological activity is a kind of life phenomenon that is produced on the basis of normal physiological activity of human body. Meanwhile, the production of psychological activity, in turn, affects various physiological activities of human body. It is on the basis of this dialectical relationship that acupuncture can affect people's various pathological and psychological processes by regulating people's physiological activities.

This study applied acupuncture combined with emotional therapy of Chinese medicine treatment during the epidemic period of COVID-19, which not only effectively lightened the mood of the elderly patients but also played a positive role in psychological suggestion, such that patients enhance the acupuncture manipulation and acupoint treatment of the psychological trust and benefit. This method can reduce the psychological craving caused by physical discomfort and emotional disorder (abnormal) in the elderly patients with alcohol dependence. During the epidemic period of COVID-19, acupuncture combined with emotional therapy of Chinese medicine treatment can improve the depressive symptoms of the elderly patients with alcohol dependence. After the outbreak of the COVID-19 epidemic, people's psychological state has also changed because of the influence of the epidemic situation and the change of lifestyle. The COVID-19 virus has the characteristics of long latent period, atypical clinical symptoms, easily missed diagnosis and misdiagnosis, and long isolation period. Due to the rapid spread of information, it is difficult for the public to distinguish true from the false information. In particular, it has caused significant short- and long-term physical and mental health damage to the elderly (Li et al., 2003; Zhu et al., 2004; Lee et al., 2006). The anxiety and depression experienced by the elderly due to the epidemic cannot be effectively addressed, resulting in an increase in the number of elderly people suffering from alcohol dependence, which requires additional attention. Therefore, it is of great significance to find an effective treatment method.

## Conclusion

By clinical observation, it was found that the depression of the elderly patients with alcohol dependence during the epidemic period of COVID-19 was not only caused by emotional stimulation but also caused by the loss of confidence and determination after the failure of abstention, a violent mood change. Because of the long-term effects of alcohol, metabolic disorders and abnormal secretion of neurotransmitters in the body cause a series of chronic pain, dizziness, and other physical discomfort and depression. Elderly patients with alcohol dependence and depression are often depressed due to emotional injury. Acupuncture can adjust the patients' emotional disorder by regulating the circulation of the body. Human's psychological activity and physiological activity are a pair of main contradictory movements in normal life. They interact and condition each other. Psychological activity is a kind of life phenomenon that is produced on the basis of normal physiological activity of human body. Meanwhile, the production of psychological activity, in turn, affects various physiological activities of human body. It is on the basis of this dialectical relationship that acupuncture can affect people's various pathological and psychological processes by regulating people's physiological activities. This study applied acupuncture combined with emotional therapy of Chinese medicine treatment during the epidemic period of COVID-19, which not only effectively lightened the mood of the elderly patients but also played a positive role in psychological suggestion, such that patients enhance the acupuncture manipulation and acupoint treatment of the psychological trust and benefit. This method can reduce the psychological craving caused by physical discomfort and emotional disorder (abnormal) in the elderly patients with alcohol dependence. During the epidemic period of COVID-19, acupuncture combined with emotional therapy of Chinese medicine treatment can improve the depressive symptoms of the elderly patients with alcohol dependence.

## Data Availability Statement

The raw data supporting the conclusions of this article will be made available by the authors, without undue reservation.

## Ethics Statement

The studies involving human participants were reviewed and approved by Heilongjiang University of Chinese medicine Ethical Commission. The patients/participants provided their written informed consent to participate in this study.

## Author Contributions

CW, FZ, and XT contributed to the conception and design of the study, contributed to the acquisition of data, contributed to the analysis and interpretation of data, contributed to the drafting of the manuscript, and contributed to the critical revision of the manuscript. All authors read and approved the final manuscript for publication.

## Conflict of Interest

The authors declare that the research was conducted in the absence of any commercial or financial relationships that could be construed as a potential conflict of interest.
